# High-sensitive cardiac troponin for the diagnosis of acute myocardial infarction in different chronic kidney disease stages

**DOI:** 10.1186/s12872-020-01746-0

**Published:** 2021-02-17

**Authors:** Daijin Ren, Tianlun Huang, Xin Liu, Gaosi Xu

**Affiliations:** Department of Nephrology, The Second Affiliated Hospital of Nanchang University, No. 1, Minde Road, Donghu District, Nanchang, 330006 People’s Republic of China

**Keywords:** Acute myocardial infarction, Chronic kidney disease, Estimated glomerular filtration rate, High-sensitivity cardiac troponin

## Abstract

**Background:**

Chronic kidney disease (CKD) are associated with acute myocardial infarction (AMI). High-sensitive cardiac troponin (hs-cTn) has been evidenced to enhance the early diagnostic accuracy of AMI, but hs-cTn levels are often chronically elevated in CKD patients, which reduces their diagnostic utility. The aim of this study was to derive optimal cutoff-values of hs-cTn levels in patients with CKD and suspected AMI.

**Methods:**

In this retrospective paper, a total of 3295 patients with chest pain (2758 in AMI group and 537 in Non-AMI group) were recruited, of whom 23.1% were had an estimated glomerular filtration rate (eGFR) of < 60 mL min^−1^ (1.73 m^2^)^−1^. Hs-cTnI values were measured at presentation.

**Results:**

AMI was diagnosed in 83.7% of all patients. The optimal value of hs-TnI in diagnosing AMI was 1.15 ng mL^−1^, which were higher in males than females comparing different cutoff-values of subgroups divided by age, gender and renal function, and which increased monotonically with decreasing of eGFR because in patients with CKD without AMI, the correlation between hs-cTnI and renal function is low but significant (r^2^ = 0.067, *P* < 0.001).

**Conclusions:**

Different optimal cutoff-values of hs-cTnI in the diagnosis of AMI in patients with CKD were helpful to the clinical diagnosis of AMI in various populations and were higher in males than females, but which was needed to be validated by multicenter randomized controlled clinical studies in the future.

## Introduction

Chronic kidney disease (CKD) is a major public health worldwide, and the most obvious outcome of which is kidney failure requiring treatment with dialysis or transplantation at a high cost [1]. However, CKD is often associated with cardiovascular disease (CVD) [1]. It has been reported that the prevalence rate among CKD individuals encountering with acute myocardial infarction (AMI) accounted for around 73.4%, and cardiovascular mortality in dialysis patients was 10–30 times higher than in the general population [1, 2]. Therefore, it is crucial to quickly diagnose AMI in patients with CKD who presenting with symptoms suggestive of Acute Coronary Syndrome (ACS).

Coronary angiography (CAG) is the gold standard for diagnosing AMI, but the widespread use of contrast media (CM) may lead to a frequently overlooked complication, contrast induced-acute kidney injury (CI-AKI), with rates ranging from 2.5 to 13.1% [3]. At the same time, high-sensitive cardiac troponin (hs-cTn) has been evidenced to improve the early diagnostic accuracy of AMI in many assays and has been widely applied in clinical practice [4], whereas elevated hs-cTn levels were observed in patients with CKD without significant myocardial necrosis, which might confuse clinicians to make an accurate and quick diagnosis when patients simultaneously presented with CKD and atypical clinical manifestation of AMI [5].

There is an urgent need to find a way to improve the diagnostic accuracy of CKD incorporating AMI instead of relying solely on CAG, and it has been attempted to improve the diagnostic accuracy of CKD with AMI by using a larger hs-cTn value to improve the diagnostic threshold, which would compensate for the loss of specificity but at the cost of decreased sensitivity [6]. In addition, the latest guide named “Fourth universal definition of myocardial infarction” has put forward that kidney disease can result in the elevation of hs-cTn, but does not mention specific data to help diagnose patients with CKD and AMI [7]. Finally, there are many other non-cardiovascular conditions-related causes influences-cTn concentration in patients suspected of AMI, such as Gender, Age, Type 2 diabetes, Sampling time and etc. [8–10].

Therefore, according to different influencing factors, we conducted the current study to enhance the diagnostic confidence of AMI in patients with CKD by getting different optimal cutoffs of high-sensitive cardiac troponin I (hs-cTnI) concertation, including the largest sample size in Asia.

## Method and materials

### Study population

**Fig. 1:**
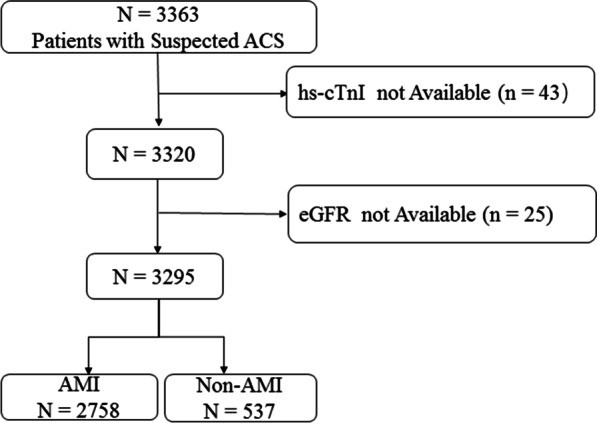
Flow charts for the inclusion of study subjects. *ACS* acute coronary syndrome, *AMI* acute myocardial infarction, *hs-cTnI* high-sensitive cardiac troponin I, *eGFR* estimated glomerular filtration rate

Clinical characteristics and laboratory test data of the Second Affiliated Hospital of Nanchang University from January 2011 to September 2017 were collected. Patients requiring renal replacement therapy were not eligible to participate. A total of 3363 patients with suspected AMI were included for the current analysis. Hs-cTnI had not be been measured in 43 patients and estimated glomerular filtration rate (eGFR) was not available in 25 patients. The final diagnosis of AMI was determined by 2 independent cardiologists based on medical records: symptoms of myocardial ischemia, patient's present and past medical history, results of laboratory testing (including hs-TnI or serum myocardial enzyme levels), image examination, electrocardiogram (ECG) (including new ST-T changes or a new Q wave) and CAG according to 2018 “Fourth universal definition of myocardial infarction” [7], as shown in Fig. 1.

### Clinical and laboratory examination

All the detailed clinical history, including age, sex, risk factors (hypertension, hypercholesterolemia, diabetes mellitus (DM), current smoking and history of smoking), coronary artery disease (CAD) history, vital status, laboratory measurements (serum creatinine, hs-cTnI) and time from symptom onset, was extracted from medical recordings.

Blood and vital status were collected directly at presentation. The hs-cTnI assay (ARCHITECT STAT hs Troponin I, Abbott Diagnostics, Abbott Park, IL) that was drawn in the medical records has an assay range of 0–50,000 ng L^−1^, with the detection limit of 1.9 ng L^−1^ and the 99th-percentile diagnostic cutoff of 30 ng L^−1^ [11]. DM was defined in our hospital in accordance with OGTT-based WHO criteria [12], and hypertension was diagnosed as systolic blood pressure (SBP) of 140 mmHg or above, or diastolic blood pressure (DBP) of 90 mmHg or above, or taking hypotensive drugs [13]. Estimated glomerular filtration rate (eGFR) was computed with CKD-EPI creatinine equation in accordance with 2012 KDIGO guidelines [14].

### Statistical methods

Continuous variables are expressed as medians (1st quartile, 3rd quartile) and categorical variables as numbers and percentages. normally distributed variables were compared with Student’s t-test and skewed distribution variables were analyzed with the Mann–Whitney U test, and categorical variables were assessed by the chi-square test. Receiver operating characteristic (ROC) curves were constructed to calculate the cutoff-value of hs-TnI to diagnose AMI, in order to improve diagnostic accuracy of hs-cTnI measured at presentation in patients with suspected AMI. Diagnostic accuracy was quantified by the area under the curve (AUC), plotted by MedCalc statistical software. Correlation between hs-cTnI and renal function was quantified by Pearson’s correlation coefficient, constructed by GraphPad Prism version 8.0. Receiver Other statistical analysis was performed with IBM SPSS Statistics for Windows, version 23.0 (Chicago, IL, USA). *P* values < 0.05 were considered statistically significant.

## Results

### Patient characteristics

In the study, 3295 patients suspected with AMI were recruited (2758 in AMI group and 537 in Non-AMI group). The baseline characteristics of patients in each group are shown in Table 1. Mean age of the patients was 61 years and 2483 (75.4%) of the participants were male. Patients in AMI group were older with higher proportions of males, hypertension, hypercholesterolemia, diabetes mellitus, family history of CAD and prior CAD than Non-AMI group (*P* < 0.001). Patients of AMI group had higher hs-TnI level compared with Non-AMI group (*P* < 0.001) and baseline hs-TnI concentrations were 20.91 (2.46, 48.03) ng mL^−1^ and 0.57 (0.16, 0.44) ng mL^−1^, respectively. There was no significant influence in current smoking or history of smoking between two groups, *P* values of which were 0.069 and 0.076, respectively. There was also no difference in time from symptom onset between two groups.

**Table 1:** Baseline characteristics of patients

	Total	AMI	Non-AMI	*P* value
Number of patients (n, %)	3295	2758 (83.7)	537 (16.3)	
Age (years)	61 (48, 71)	64 (55, 74)	57 (43, 70)	< 0.001
Sex (male)	2483 (75.4)	2165 (78.5)	318 (59.2)	< 0.001
Risk factors				
Hypertension	1688 (51.2)	1567(56.8)	121 (22.5)	< 0.001
Hypercholesterolemia	930 (28.2)	880 (31.9)	50 (9.3)	< 0.001
Diabetes mellitus	682 (20.7)	646 (23.4)	26 (4.8)	< 0.001
Current smoking	1519 (46.1)	1288 (46.7)	231 (43.2)	0.069
History of smoking	1770 (53.7)	1497(54.3)	273 (50.8)	0.076
History				
Family history of CAD	82 (2.6)	77 (2.8)	5 (0.87)	< 0.001
Prior CAD	758 (23.0)	723 (26.2)	35 (6.5)	< 0.001
Vital status				
Heart rate (bpm)	76 (64, 88)	79 (66, 94)	74 (63, 84)	< 0.001
Systolic blood pressure (mmHg)	125 (115, 137)	143 (129, 162)	119 (114, 132)	< 0.001
Diastolic blood pressure (mmHg)	81 (71, 93)	85 (76, 94)	73 (68, 82)	< 0.001
Hs-cTnI (ng mL^−1^)	19.43 (1.38, 48.60)	20.91 (2.46, 48.03)	0.57 (0.16, 0.44)	< 0.001
Time from symptom onset (h)				
< 6 (n, %)	1267 (38.5)	1063 (38.5)	204 (38.0)	0.977
≥ 6 (n, %)	2028 (61.5)	1695 (61.5)	333 (62.0)	0.976

For group comparisons, Student’s *t* test was used for normally distributed variables and non-parametric Mann–Whitney tests for non-normally distributed variables. Categorical variables were compared with a χ^2^-test

*AMI* acute myocardial infarction, *CAD* coronary artery disease, *Hs-cTnI* high-sensitive cardiac troponin I

### Hs-cTnI and renal function

**Fig. 2:**
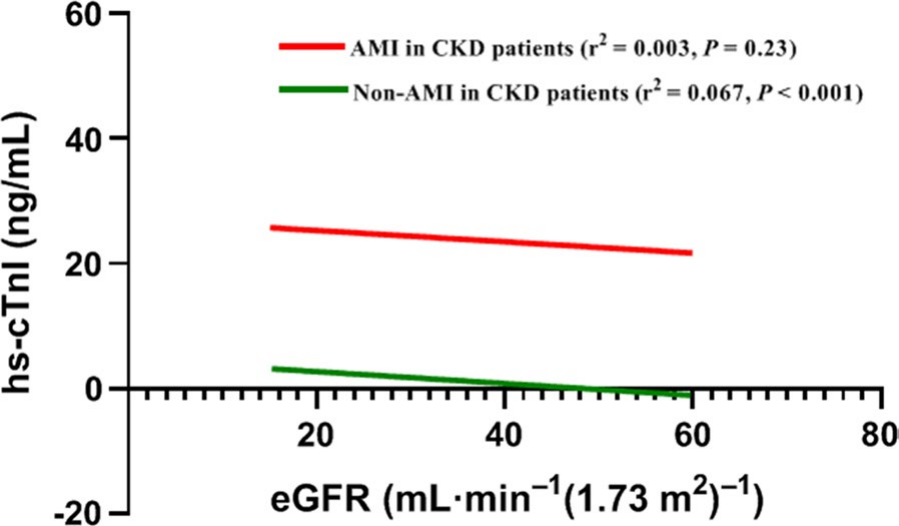
The association of hs-cTnI with measures of renal function in patients with CKD [eGFR < 60 mL min^−1^ (1.73 m^2^)^−1^] by Pearson’s correlation coefficient, and compared hs-cTnI levels between patients with or without AMI. There was a low but significant correlation between hs-cTnI and renal function in CKD patients without AMI (r^2^ = 0.067, *P* < 0.001), but there was no correlation in CKD patients with AMI (r^2^ = 0.003, *P* = 0.23). Furthermore, we found that CKD could result in the elevation of hs-cTnI without AMI. *AMI* acute myocardial infarction, *eGFR* estimated glomerular filtration rate, *hs-cTnI* high-sensitive cardiac troponin I, *CKD* chronic kidney disease

We assessed the association of hs-cTnI with measures of renal function in patients with CKD [eGFR < 60 mL min^−1^ (1.73 m^2^)^−1^] by Pearson’s correlation coefficient, and compared hs-cTnI levels between patients with or without AMI (as shown in Fig. 2). There was a low but significant correlation between hs-cTnI and renal function in CKD patients without AMI (r^2^ = 0.067, *P* < 0.001), but there was no correlation in CKD patients with AMI (r^2^ = 0.003, *P* = 0.23). Furthermore, we found that CKD could result in the elevation of hs-cTnI without AMI.

Based on the above, we concluded that in presenting with CKD and non-AMI patients, the lower the eGFR, the higher the hs-cTnI concentration, but in AMI individuals, which was not.

### Diagnostic accuracy of hs-cTnI at presentation

**Fig. 3:**
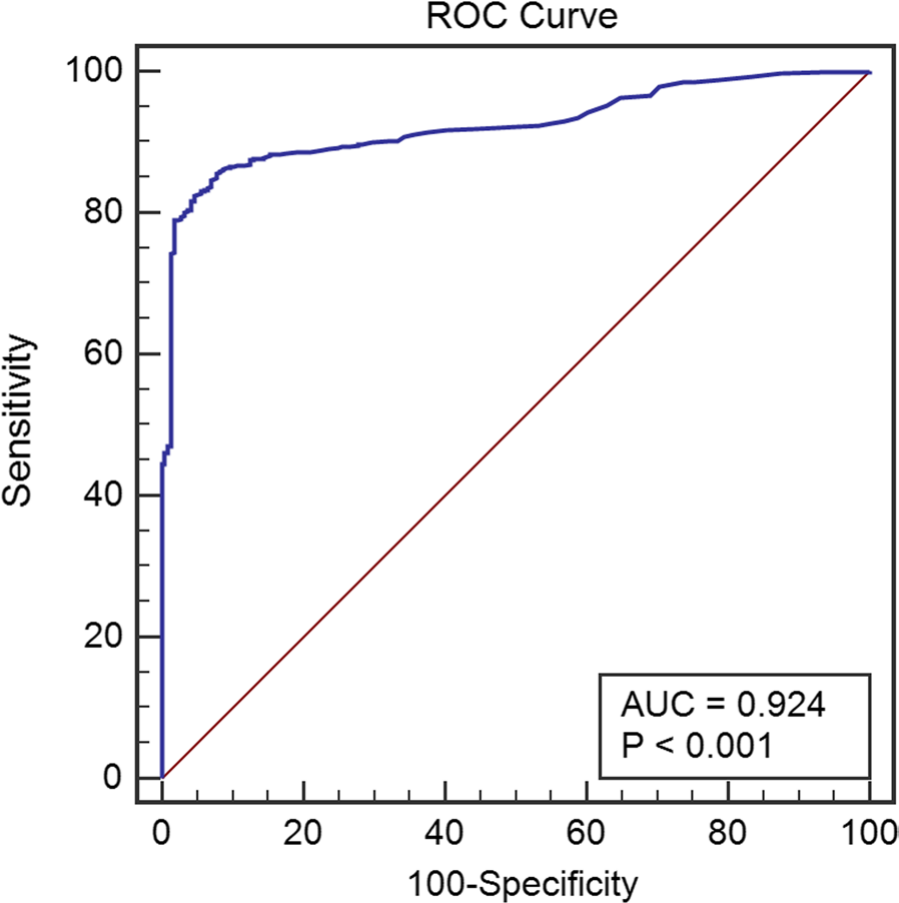
ROC curve for diagnosis of AMI in the total study population. The optimal cutoff-value for diagnosis of AMI in all suspicious patients, which appeared to be an hs-TnI level of 1.15 ng mL^−1^ with 82.5% sensitivity and 95.4% specificity. AUC was 0.924 [95% CI (0.914, 0.933)]. *AMI* acute myocardial infarction, *hs-cTnI* high-sensitive cardiac troponin I; *AUC* area under the curve, *ROC* receiver-operating characteristic

**Fig. 4:**
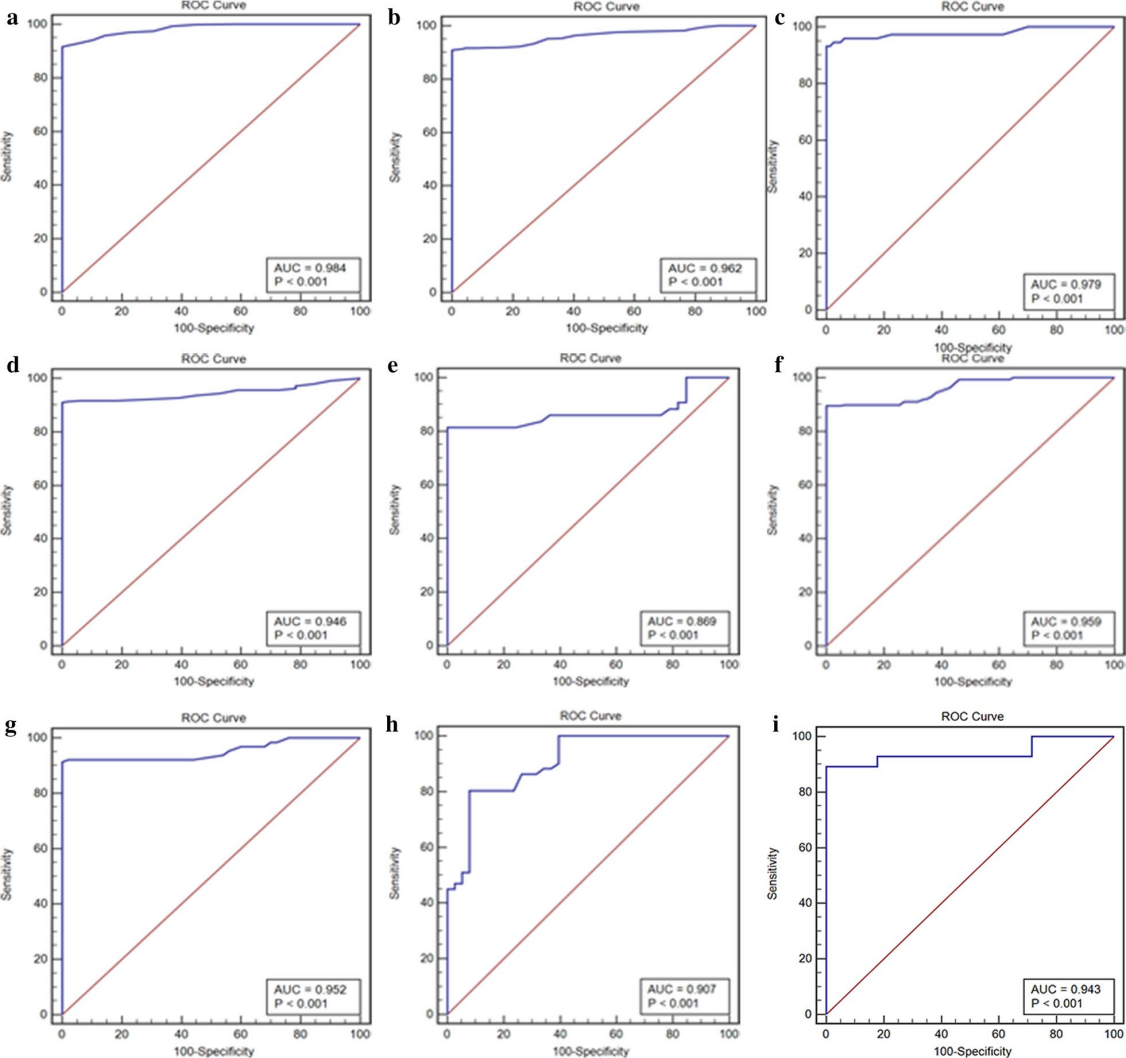
ROC curve for diagnosis of AMI divided by age, gender and renal function. **a** Male patients younger than 60 years with eGFR ≥ 60 mL min^−1^ (1.73 m^2^)^−1^, Cut-off = 0.24 ng mL^−1^ with 91.6% sensitivity and 100.0% specificity. AUC = 0.984 [95% CI (0.973, 0.991)]. **b** Male patients aged 60 or above with eGFR ≥ 60 mL min^−1^ (1.73 m^2^)^−1^, Cut-off = 0.31 ng mL^−1^ with 90.9% sensitivity and 100.0% specificity. AUC = 0.962 [95% CI (0.949, 0.973)]. **c** Female patients younger than 60 years with eGFR ≥ 60 mL min^−1^ (1.73 m^2^)^−1^, Cut-off = 0.27 ng mL^−1^ with 93.2% sensitivity and 100.0% specificity. AUC = 0.979 [95% CI (0.941, 0.995)]. **d** Female patients aged 60 or above with eGFR ≥ 60 mL min^−1^ (1.73 m^2^)^−1^, Cut-off = 0.27 ng mL^−1^ with 91.0% sensitivity and 100.0% specificity. AUC = 0.946 [95% CI (0.918, 0.966)]. **e** Male patients younger than 60 years with 30 ≤ eGFR < 60 mL min^−1^·(1.73 m^2^)^−1^, Cut-off = 0.72 ng mL^−1^ with 81.4% sensitivity and 100.0% specificity. AUC = 0.869 [95% CI (0.772, 0.936)]. **f** Male patients aged 60 or above with 30 ≤ eGFR < 60 mL min^−1^·(1.73 m^2^)^−1^, Cut-off = 0.50 ng mL^−1^ with 89.5% sensitivity and 100.0% specificity. AUC = 0.959 [95% CI (0.932, 0.978)]. **g** Female patients aged 60 or above with 30 ≤ eGFR < 60 mL min^−1^·(1.73 m^2^)^−1^, Cut-off = 0.59 ng mL^−1^ with 91.4% sensitivity and 100.0% specificity. AUC = 0.952 [95% CI (0.910, 0.979)]. **h** Male patients aged 60 or above with 15 ≤ eGFR < 30 mL min^−1^ (1.73 m^2^)^−1^, Cut-off = 2.61 ng mL^−1^ with 80.4% sensitivity and 92.1% specificity. AUC = 0.907 [95% CI (0.827, 0.958)]. **i** Female Patients aged 60 or above with 15 ≤ eGFR < 30 mL min^−1^ (1.73 m^2^)^−1^, Cut-off = 1.90 ng mL^−1^ with 89.3% sensitivity and 92.1% specificity. AUC = 0.943 [95% CI (0.846, 0.987)]. *AMI* acute myocardial infarction, *hs-cTnI* high-sensitive cardiac troponin I, *AUC* area under the curve, *ROC* receiver-operating characteristic, *eGFR* estimated glomerular filtration rate

By constructing ROC curve, we got the optimal cutoff-value for diagnosis of AMI in all suspicious patients, which appeared to be an hs-TnI level of 1.15 ng mL^−1^ with 82.5% sensitivity and 95.4% specificity. AUC was 0.924 [95% CI (0.914, 0.933)] (Fig. 3). Then, we grouped those patients by different age, sex, and eGFR categories (Table 2 and Fig. 4), and calculated their optimal cutoff-values of hs-TnI levels to improve diagnostic accuracy of AMI in CKD patients, respectively.

**Table 2:** Diagnostic accuracy of hs-cTnI for AMI in patients according to sex, age and eGFR category

	AMI (n, %)	Sensitivity (%, 95% CI)	Specificity (%, 95% CI)	AUC (95% CI)	*P* value
All patients (n = 3295)	2758 (83.7)				
Cut-off = 1.15 ng mL^−1^		82.5 (81.0, 83.9)	95.4 (91.7, 97.8)	0.924 (0.914, 0.933)	< 0.001
(a) Male patients younger than 60 years with eGFR ≥ 60 mL min^−1^ (1.73 m^2^)^−1^ (n = 896)	798 (89.1)				
Cut-off = 0.24 ng mL^−1^		91.6 (89.5, 93.4)	100.0 (96.3, 100.0)	0.984 (0.973, 0.991)	< 0.001
(b) Male patients aged 60 or above with eGFR ≥ 60 mL min^−1^ (1.73 m^2^)^−1^ (n = 1088)	1028 (94.5)				
Cut-off = 0.31 ng mL^−1^		90.9 (88.9, 92.6)	100.0 (95.9, 100.0)	0.962 (0.949, 0.973)	< 0.001
(c) Female patients younger than 60 years with eGFR ≥ 60 mL min^−1^ (1.73 m^2^)^−1^ (n = 154)	81 (52.6)				
Cut-off = 0.27 ng mL^−1^		93.2 (84.9, 97.8)	100.0 (95.5, 100.0)	0.979 (0.941, 0.995)	< 0.001
(d) Female patients aged 60 or above with GFR ≥ 60 mL min^−1^ (1.73 m^2^)^−1^ (n = 396)	357 (90.2)				
Cut-off = 0.27 ng mL^−1^		91.0 (87.5, 93.8)	100.0 (93.0, 100.0)	0.946 (0.918, 0.966)	< 0.001
(e) Male patients younger than 60 years with 30 ≤ eGFR< 60 mL min^−1^ (1.73 m^2^)^−1^ (n = 76)	56 (73.7)				
Cut-off = 0.72 ng mL^−1^		81.4 (66.6, 91.6)	100.0 (89.4, 100.0)	0.869 (0.772, 0.936)	< 0.001
(f) Male patients aged 60 or above with 30 ≤ eGFR< 60 mL min^−1^ (1.73 m^2^)^−1^ (n = 330)	267 (80.9)				
Cut-off = 0.50 ng mL^−1^		89.5 (85.2, 92.9)	100.0 (94.3, 100.0)	0.959 (0.932, 0.978)	< 0.001
(g) Female patients aged 60 or above with 30 ≤ eGFR< 60 mL min^−1^ (1.73 m^2^)^−^1 (n = 178)	128 (71.9)				
Cut-off = 0.59 ng mL^−1^		91.4 (85.1, 95.6)	100.0 (92.9, 100.0)	0.952 (0.910, 0.979)	< 0.001
(h) Male patients aged 60 or above with 15 ≤ eGFR< 30 mL min^−1^ (1.73 m^2^)^−1^ (n = 89)	51 (57.3)				
Cut-off = 2.61 ng mL^−1^		80.4 (66.9, 90.2)	92.1 (78.6, 98.3)	0.907 (0.827, 0.958)	< 0.001
(i) Female patients aged 60 or above with 15 ≤ eGFR< 30 mL min^−1^ (1.73 m^2^)^−1^ (n = 56)	28 (50.0)				
Cut-off = 1.90 ng mL^−1^		89.3 (71.8, 97.7)	100.0 (87.7, 100.0)	0.943 (0.846, 0.987)	< 0.001

*AMI* acute myocardial infarction, *eGFR* estimated glomerular filtration rate, *hs-cTnI* high-sensitive cardiac troponin I, *CI* confidence interval, *AUC* area under the curve

In the “Male Patients younger than 60 years with eGFR ≥ 60 mL min^−1^ (1.73 m^2^)^−1^” group, there were 896 patients. AMI was the adjudicated final diagnosis in 89.1% of them. The optimal cutoff-value for diagnosis of AMI was 0.24 ng mL^−1^ with 91.6% sensitivity and 100.0% specificity. AUC was 0.984 [95% CI (0.973, 0.991)] (Fig. 4a).

In the “Male Patients aged 60 or above with eGFR ≥ 60 mL min^−1^ (1.73 m^2^)^−1^” group, there were 1088 patients. AMI was the adjudicated final diagnosis in 94.5% of them. The optimal cutoff-value for diagnosis of AMI was 0.31 ng mL^−1^ with 90.9% sensitivity and 100.0% specificity. AUC was 0.962 [95% CI (0.949, 0.973)] (Fig. 4b).

In the “Female Patients younger than 60 years with eGFR ≥ 60 mL min^−1^ (1.73 m^2^)^−1^” group, there were 154 patients. AMI was the adjudicated final diagnosis in 52.6% of them. The optimal cutoff-value for diagnosis of AMI was 0.27 ng mL^−1^ with 93.2% sensitivity and 100.0% specificity. AUC was 0.979 [95% CI (0.941, 0.995)] (Fig. 4c).

In the “Female Patients aged 60 or above with eGFR ≥ 60 mL min^−1^ (1.73 m^2^)^−1^” group, there were 396 patients. AMI was the adjudicated final diagnosis in 90.2% of them. The optimal cutoff-value for diagnosis of AMI was 0.27 ng mL^−1^ with 91.0% sensitivity and 100.0% specificity. AUC was 0.946 [95% CI (0.918, 0.966)] (Fig. 4d).

In the “Male Patients younger than 60 years with 30 ≤ eGFR < 60 mL min^−1^ (1.73 m^2^)^−1^” group, there were 76 patients. AMI was the adjudicated final diagnosis in 73.7% of them. The optimal cutoff-value for diagnosis of AMI was 0.72 ng mL^−1^ with 81.4% sensitivity and 100.0% specificity. AUC was 0.869 [95% CI (0.772, 0.936)] (Fig. 4e).

In the “Male Patients aged 60 or above with 30 ≤ eGFR < 60 mL min^−1^ (1.73 m^2^)^−1^” group, there were 330 patients. AMI was the adjudicated final diagnosis in 80.9% of them. The optimal cutoff-value for diagnosis of AMI was 0.50 ng mL^−1^ with 89.5% sensitivity and 100.0% specificity. AUC was 0.959 [95% CI (0.932, 0.978)] (Fig. 4f).

In the “Female Patients aged 60 or above with 30 ≤ eGFR < 60 mL min^−1^ (1.73 m^2^)^−1^” group, there were 178 patients. AMI was the adjudicated final diagnosis in 71.9% of them. The optimal cutoff-value for diagnosis of AMI was 0.59 ng mL^−1^ with 91.4% sensitivity and 100.0% specificity. AUC was 0.952 [95% CI (0.910, 0.979)] (Fig. 4g).

In the “Male Patients aged 60 or above with 15 ≤ eGFR < 30 mL min^−1^ (1.73 m^2^)^−1^” group, there were 89 patients. AMI was the adjudicated final diagnosis in 57.3% of them. The optimal cutoff-value for diagnosis of AMI was 2.61 ng mL^−1^ with 80.4% sensitivity and 92.1% specificity. AUC was 0.907 [95% CI (0.827, 0.958)] (Fig. 4h).

In the “Female Patients aged 60 or above with 15 ≤ eGFR < 30 mL min^−1^ (1.73 m^2^)^−1^” group, there were 56 patients. AMI was the adjudicated final diagnosis in 50.0% of them. The optimal cutoff-value for diagnosis of AMI was 1.90 ng mL^−1^ with 89.3% sensitivity and 100.0% specificity. AUC was 0.943 [95% CI (0.846, 0.987)] (Fig. 4i).

However, in the “Female Patients younger than 60 years with 30 ≤ eGFR < 60 mL min^−1^ (1.73 m^2^)^−1^” group, there were only 18 patients and 4 patients were diagnosed as AMI, hs-cTnI data of which was 10.04 (0.28, 11.36) ng mL^−1^. In the “Male Patients younger than 60 years with 15 ≤ eGFR < 30 mL min^−1^ (1.73 m^2^)^−1^” group, there were only 9 patients and 5 patients were diagnosed as AMI, hs-cTnI data of which was 8.88 (0.90, 2.60) ng mL^−1^. In the “Female Patients younger than 60 years with 15 ≤ eGFR < 30 mL min^−1^ (1.73 m^2^)^−1^” group, there were only 5 patients and 1 patients were diagnosed as AMI, hs-cTnI data of which was 0.49 (0.46, 0.51) ng mL^−1^. Considering these groups with small sample sizes, we did not perform ROC curve with them.

## Discussion

Patients with CKD are often associated with CVD, especially, AMI with atypical symptoms [15], who often do not receive the best possible treatment for AMI, including antithrombotic drugs and coronary angiography. It was hard for clinicians to make the correct diagnosis of AMI because patients with CKD were more insensitive to chest pain and hs-cTnI levels often rise chronically even in the absence of AMI [16, 17]. And the latest universal definition of AMI also referred that many patients with chronic kidney disease (CKD) have elevation of hs-cTnI values [7], but which did not provide for optimal cutoffs of it. Therefore, to improve the diagnostic accuracy of AMI in patients with CKD, it is necessary to calculate cutoffs of hs-cTnI levels.

Some studies had tested this cutoff value in patients with different renal functions [10, 18]. A prospective multicenter diagnostic study enrolling individuals with suspected non-ST-segment elevation myocardial infarction, might better help diagnose atypical symptoms of AMI by computing different cut-off values of hs-cTnI and raising the 0/1-h algorithm, but which with high sensitivity and lower specificity easily leading to misdiagnosis, and which was unrealistic to observe troponin changes dynamically by repeated blood, because once the suspected patient went to the hospital, the coronary angiography should be performed immediately to assist the diagnosis, so that the troponin cannot be taken again and patients with renal insufficiency were more likely to be complicated with anemia so that repeated blood collection might aggravate the anemia [10]. In addition, another similar study also showed the above shortcomings by calculating different cut-off values of hs-cTnI and raising the 0/3-h algorithm [18]. Furthermore, two retrospective studies computed different cut-off values of hs-cTn levels according to eGFR category, which also presenting with high sensitivity and lower specificity [4, 19], but there were other factors influencing hs-cTnI concentrations as well as the triage of patients with suspected AMI. Some studies indicated that age was the most important non-cardiac determinant of hs-cTnI levels [20, 21], and patients aged over 60 years had higher hs-cTnI levels [22]. Gender was also an important factor and the median hs-cTnI concentration was significantly lower in women. It has been suggested that the under-diagnosis of AMI in women was due to the inappropriate diagnostic thresholds and gender-specific thresholds were recommended for hs-cTnI assays to improve the diagnostic accuracy [23].

In this retrospective study involving 3295 patients, 23.1% of whom were CKD [15 ≤ eGFR < 60 mL min^−1^ (1.73 m^2^)^−1^] excluding patients of end-stage kidney disease, constructed the diagnostic performance of an hs-cTnI assay taken at presentation in patients presenting with acute chest pain. We compared hs-TnI levels in AMI and non-AMI patients, as well as in the subgroups of different age, sex and renal function categories. And we made a simple linear regression analysis to simulate the association of hs-cTnI with renal function. Further, we performed ROC curves to compute optimal hs-TnI levels for helping diagnose of AMI in different categories individuals.

To our acknowledgment, our study is the first analysis to calculate optimal cutoff-values to improve the diagnostic accuracy of AMI in patients with CKD, based on different age, gender and renal kidney subgroups, which may promote early diagnosis and intervention of AMI further to improve prognosis in CKD patients.

Our study has shown that the correlation of hs-cTnI with eGFR are weak but significant in CKD patients [15 ≤ eGFR < 60 mL min^−1^ (1.73 m^2^)^−1^] without AMI patients. That is to say, the cutoff-values of hs-cTnI concentrations were increased monotonically with decreasing of eGFR, but in CKD with AMI individuals, which were not. This outcome was in accordance with the literature, which has been made an explanation that CKD patients were more insensitive to chest pain, leading to missed diagnosis of symptomLess AMI patients with CKD [19], on the other hand, we also made another explanation, which was many patients diagnosed with AMI have reached the highest concentration of hs-cTnI (50 ng mL^−1^) that could be detected in our laboratory, at the same time, hs-cTnI levels were independent of changes in eGFR. And we performed 9 ROC curves based on subgroups divided by age, gender and eGFR to get different cutoff-values of hs-cTnI concentrations, and 7 of these groups’ specificity reached to 100%, which were significantly higher than other similar studies [4, 10, 18, 19]. Our data also showed hs-cTnI concentrations were higher in males than females, comparing different genders in the same age and kidney function group, which was in agreement with the literature [22]. Further, the cutoff-value of hs-cTnI concentrations in patients aged 60 or above was not higher than below 60 years, comparing different age stages in the same gender and kidney function group in disagreement with the literature [23], which might be associated with some small sample sizes’ groups that could not be calculated cutoff-values.

However, some limitations in this study are as follows: Firstly, there were small sample sizes subgroups that have been excluded to calculate cutoff-values, so that we could not properly compare the influence of different risk factors on troponin values. Secondly, we did not include the particular of uremia patients with minority. Thirdly, there were other hs-cTn assays, but in this study, we only applied hs-TnI assay to comment on the clinical utility in CKD individuals. Fourthly, we just performed subgroups analysis according to age, gender and renal function because of the limitation of sample size, but there were other related factors that could influence hs-cTn concentration, such as Type 2 diabetes, Hypertension, Neuromuscular diseases and so on [9, 24, 25]. New studies including larger sample size should be performed and grouped by more factors as mentioned above. Fifthly, we could not compare the optimal cut-off values according to different age stages. Lastly, this retrospective study was conducted based on medical records from a single center. There should be more multicenter prospective randomized controlled trials to evidence.

## Conclusion

Different optimal cutoff-values of hs-cTnI for diagnosis of AMI in CKD patients were useful for clinical diagnosis of AMI in various populations and were higher in males than females, and 7 of 9 subgroups’ specificity reached to 100%, but which were needed to be validated by multicenter randomized controlled clinical studies in the future.

## Data Availability

The datasets generated during the current study are not publicly available because which involves confidential patient data but are available from the corresponding author on reasonable request.

## Abbreviations

AMI: Acute myocardial infarction
CKD: Chronic kidney disease
eGFR: Estimated glomerular filtration rate
CAD: Coronary artery disease
Hs-cTnI: High-sensitive cardiac troponin I
CI: Confidence interval
AUC: Area under the curve
ROC: Receiver-operating characteristic
ACS: Acute Coronary Syndrome
CAG: Coronary angiography
CVD: Cardiovascular disease
CM: Contrast media
CI-AKI: Contrast induced-acute kidney injury
DM: Diabetes mellitus
SBP: Systolic blood pressure
DBP: Diastolic blood pressure
